# The role of common mental disorders on sustainable working life—a cohort study among discordant Swedish twin pairs

**DOI:** 10.1136/bmjopen-2025-101586

**Published:** 2025-11-04

**Authors:** Annina Ropponen, Iman Alaie, Jurgita Narusyte, Pia Svedberg

**Affiliations:** 1 CNS, Division of Insurance Medicine, Karolinska Institutet, Stockholm, Sweden; 2 Finnish Institute of Occupational Health, Helsinki, Finland; 3 Division of Clinical Psychology, Department of Psychology, Uppsala University, Uppsala, Sweden

**Keywords:** MENTAL HEALTH, EPIDEMIOLOGY, GENETICS, Health Workforce, OCCUPATIONAL & INDUSTRIAL MEDICINE, PUBLIC HEALTH

## Abstract

**Abstract:**

**Objectives:**

To investigate patterns of sustainable working life, defined as a few or no interruptions from paid work due to sickness absence, unemployment or disability pension among Swedish twins with and without common mental disorders (CMDs). We also sought to examine the role of baseline sociodemographic factors for the identified patterns.

**Design:**

Prospective cohort study.

**Setting:**

Population-based sample of twins born in Sweden.

**Participants:**

The sample of 5529 CMDs, discordant twin pairs between ages 18 and 59 years at baseline in 1998 (50% women) were followed annually for working life statuses using data obtained from national registers until 2020.

**Primary outcome:**

Sustainable working life.

**Measures:**

Group-based trajectory modelling was applied to identify distinct trajectory groups. Multinomial logistic regression models estimating ORs were performed.

**Results:**

For those with CMDs, a three-trajectory solution was the best-fitting model, while for those without CMDs, a two-trajectory solution had best fit; in both groups, sustainable working life constituted the largest trajectory group (71% and 83%, respectively). No sustainable working life yielded 14.5% and 17% in those with CMDs and those without CMDs, respectively, whereas, among those with CMDs, another 14.5% had a trajectory with decreasing sustainable working life. Higher education was associated with a lower likelihood (OR 0.12–0.47) and being single (with or without children, OR 2.23–2.51) with a higher likelihood of belonging to those trajectories characterised by no sustainable working life.

**Conclusion:**

A small cluster among those with CMDs tended to follow a decreasing sustainable working life pattern, while a minority with or without CMDs had no sustainable working life. Although a sustainable working life seems common, those with CMDs should be identified early for preventive actions and support to remain in paid work.

**STRENGTHS AND LIMITATIONS OF THIS STUDY**

- Large, good quality national register data of sustainable working life, mental health diagnoses and other influential factors.
- A population-based sample of 18–59-year-old Swedish-born twins discordant for the common mental disorders (CMDs) was followed from the baseline in 1998 until 2020.
- Group-based trajectory modelling using a matched discordant twin cohort for identifying the patterns of sustainable working life.
- The first incident of CMDs is limited to estimating the timing and recurring events that should be accounted for with different designs.

## Background

 Mental ill-health rates have been increasing in Sweden during the last decade.^1^ Mental ill-health rates are also high globally, since approximately 15% of working-age adults have a mental disorder.^2^ Among all mental disorders, common mental disorders (CMDs), such as depression, anxiety and stress-related conditions, are among the most prevalent globally.^3^ CMDs affect people across the lifespan, with the first onset frequently occurring in adolescence or young adulthood and oftentimes with recurring episodes later.^4^ Psychiatric and somatic comorbidities are also common among people with CMDs (eg, substance use disorders and musculoskeletal disorders).^4^,^6^ In the long run, CMDs can significantly reduce the work capacity of an individual, as suggested by the high sickness absence (SA) rates in Sweden.^7^ However, many people with CMDs are in paid work and are likely to benefit from working.^8^

CMDs are important to consider from a broader working life perspective, as good mental health is reasonably expected to support more productive work.^9^ Thus, understanding how working life participation differs between those with and without CMDs across a life course can help target interventions and preventive measures more effectively. To date, most research on CMDs among working-age populations has focused on specific working life outcomes, for example, SA,^10 11^ return-to-work^12 13^ or focusing on a specific occupational sector.^14^ Recent findings suggest that CMDs may shorten participation in working life.^15 16^ Therefore, it is essential to gain further insight into the developmental and longitudinal patterns of working life participation, comparing individuals with and without CMDs. A trajectory-based approach would allow us to estimate the proportion of individuals following specific working life paths over time.^17^

A sustainable working life can be defined as no or few interruptions due to unemployment, SA or disability pension (DP).^18^ The fit between work and individual characteristics—such as age, sex, level of education and marital status during the life course—should be emphasised, as these factors influence working life participation.^19^ From a life course perspective, several aspects warrant attention. The likelihood of SA/DP increases with age,^20 21^ and mental ill-health in childhood or adolescence is associated not only with SA/DP^22^ but also with poor participation in work.^23^ Additionally, unemployment in early adulthood predicts later SA/DP.^24^ These findings highlight the need for longitudinal research to better understand the development of sustainable working life among individuals with CMDs.

Twin studies offer a unique opportunity to assess familial factors, including genetics and shared environmental factors, mainly in childhood—key elements in understanding the aetiology of CMDs and sustainable working life. Both CMDs (eg, depression,^25^ anxiety,^26^ stress-related disorders^27^ and sustainable working life)^28^ carry a genetic component. Thus, genetics may partly explain the associations between CMDs and sustainable working life, as suggested by previous studies on SA/DP.^29^,^31^ Twins raised together share early rearing environments (eg, parental factors, socioeconomic status and home environment). Genetically, monozygotic (MZ) twin pairs are virtually identical on the gene sequence level, while dizygotic (DZ) twin pairs share about 50% of their segregating genes. Selecting discordant twin pairs—those differing in a factor of interest—provides an optimally matched sample in terms of age, sex, genetics and early environment.^32^ Applying a data-driven method^17^ to a discordant twin sample of sustainable working life can offer epidemiological insights beyond those gained from unrelated population-based samples, particularly regarding the role of genetics. Even if genetics contributes to CMDs and sustainable working life, such knowledge can help tailor interventions to individual vulnerabilities.

In this study, we aimed to investigate patterns of sustainable working life among Swedish twins discordant for CMDs using detailed register data with group-based trajectory modelling (GBTM). Another aim was to estimate the role of baseline sociodemographic and familial factors for the data-driven identified trajectory groups.

## Sample and methods

### Study design

his study is part of the Swedish Twin project of Disability pension and Sickness absence (STODS) that includes the twins identified in the Swedish Twin Registry (STR) who were born between 1925 and 1990, which included 119,907 twin individuals.^18^ We used a prospective cohort design with CMD-discordant twins from 1998 to 2020 for follow-up using register data.

### Study population

**Figure 1:**
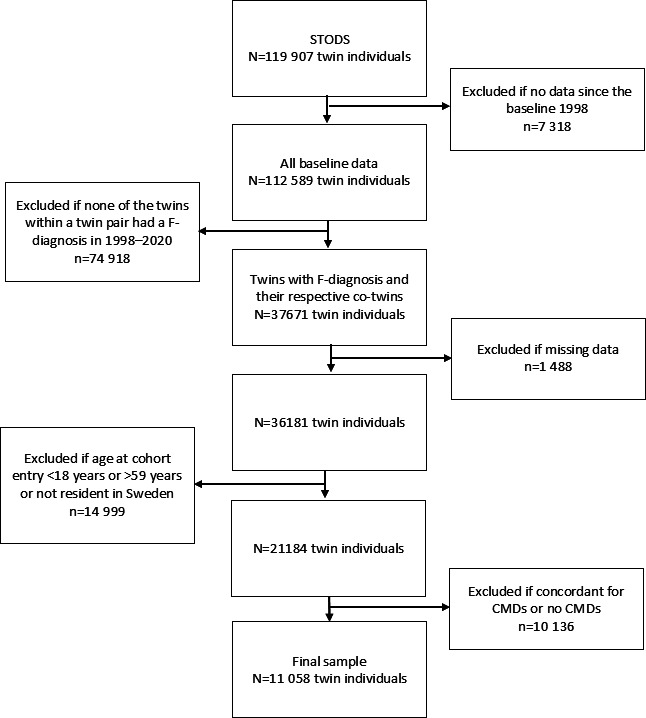
Flowchart of sample selection. CMDs, common mental disorders; STODS, Swedish Twin project of Disability pension and Sickness absence.

The sample, comprising all data at the harmonised baseline in 1998, consisted of 112,589 twin individuals (figure 1). The study population was then limited to individuals with the first incident International Classification of Diseases (ICD)-10 codes F00–F99 diagnosis (mental and behavioural disorders in any diagnosis field, ie, being the main or any other diagnosis) in inpatient care or specialised outpatient care during the study period from 1998 to 2020 and their co-twins regardless of whether they had F00–F99 diagnosis or not (n=37,671). Then we restricted the sample to those (n=36,183) without any missing data in any outcomes or factors of interest. The sample with age at cohort entry 18–59 years and all residents in Sweden at baseline and during the entire study period was 21,184, including 39 twins whose zygosity information was missing (<0.1% of the final sample). Thus, to further limit the sample, we focused on those with CMDs for follow-up and compared them to their co-twins within a twin pair without CMDs (ie, no current or later CMD diagnosis). For this, we chose only those with CMDs defined as having the diagnosis for major depressive disorders (ICD-10 code: F32 and F33), phobic anxiety disorders (ICD-10: F40), other anxiety disorders (ICD-10: F41), obsessive-compulsive disorders (ICD-10: F42) and reaction to severe stress, and adjustment disorders (ICD-10: F43), in inpatient care or specialised outpatient care (n=6577 individuals later referred as inpatient and specialised outpatient care cases of CMDs). CMDs were chosen since all diagnoses in F00–F99 represent a broad heterogeneity, while depression and anxiety largely share the aetiology and are highly comorbid.^33^ Then the sample was further limited to only discordant pairs, including only twins whose co-twin in the twin pair was without CMDs (n=11,058 individuals, figure 1). The evaluation of the role of zygosity was further limited to MZ and same-sex DZ-discordant twin pairs for CMDs: 1018 MZ and 1045 DZ same-sex pairs. To indicate the potential effect of other mental diagnoses among those twins without CMDs, we checked the first incident F00–F99 during the follow-up and categorised the diagnoses into three categories: ‘neurodevelopmental’ including F70–F79, F84 and F90 (ie, attention-deficit hyperactivity disorder, autism spectrum disorder and intellectual disabilities), ‘severe mental illness’ with F20, F23.1, F23.2, F25, F28, F30 and F31 representing psychiatric conditions like schizophrenia, schizoaffective disorder, bipolar disorder, and ‘substance use’, with F10–F19 for substance use disorders. The frequencies are shown in online supplemental table S1. We also controlled the F-diagnoses using data from inpatient care before baseline (1998, online supplemental table S2).

### Measurements

STODS contains national register data from the Micro Data for Analyses of Social Insurance of the Swedish Social Insurance Agency for information on SA and DP; from the Longitudinal Integrated Database for Health Insurance and Labour Market Studies of Statistics Sweden^34^ for unemployment, old-age pension and sociodemographic information (educational level, degree of urbanisation and marital status); and from The Causes of Death Register of the Swedish Board of Health and Welfare provided dates of death, and inpatient and specialised outpatient care diagnosis codes (ICD-10).

The outcome of this study was the degree of sustainable working life estimated using the main labour market status in each year of follow-up, based on the definitions used before:^35^ SA/DP (>180 days with SA or DP benefits from the Swedish Social Insurance Agency); unemployment (>180 days with unemployment benefits); old-age pension (more than half of yearly income from the old-age pension); or employment (ie, in paid work and did not fulfil the criteria for SA/DP, unemployment or old-age pension) as applied in earlier studies.^28 35 36^ For statistical analyses, these statuses were coded for each year being in paid work and did not fulfil the criteria for SA/DP, unemployment or old-age pension (as a proxy for employment) as ‘1’, and all other statuses as ‘0’. For censoring, we accounted for emigration and death, and the follow-up was terminated when an individual reached the age of 65 years. We used the age of 65 years as the end of the follow-up because in Sweden, there is no statutory pension age. However, most will retire at the age of 65 years.

Factors of interest were zygosity (MZ and same-sex DZ), age and sex from STR. We also used educational level (elementary school (<10 years); high school (10–12 years); university/college (>12 years); and missing), and marital status (married or cohabitant without children; married or cohabitant with children; single without children; and single with children).^34 37^ Residential regions were classified into three groups according to Swedish municipalities, based on the degree of urbanisation^38^ (cities; towns and suburbs; and rural areas). We accounted for any mental diagnosis (F00–F99) or musculoskeletal diagnosis (MSD, M00–M99) that existed before the baseline in 1998. Prior mental diagnosis or MSD was coded as yes/no. Online supplemental table S2 includes the F-diagnosis for prior mental diagnosis.

### Statistical analyses

We conducted statistical analyses using Stata 17.1 MP. The descriptive statistics were calculated using frequencies with percentages (%) for sample characteristics based on factors of interest, and among those with or without CMDs. Then we estimated trajectories for annual statuses of sustainable working life, which included employment, unemployment, SA/DP and old-age pension, across follow-up from 1998 to 2020 using GBTM.^17^ The GBTM method was used to identify trajectory groups consisting of groups of individuals that follow a distinct pattern over time. Modelling was repeated with an increased number of trajectories if the model could converge while applying a linear polynomial model. To determine the best-fitting trajectory model, we used the Bayesian information criterion (BIC), Akaike information criteria and average posterior probability (APP) as suggested previously,^17^ with BIC emphasised for the decision and a holding limit of 5% for the smallest group size. The linear polynomial was chosen over cubic or quadratic polynomials as the APP of assignment was >70% and the odds of correct classification >5 for each class.^39 40^ Based on the sample definition, we estimated the GBTM using a twin sample that was perfectly matched for age, sex and familial factors as being MZ and DZ same-sexed twins, while the trajectories were modelled separately for twins who were inpatient and specialised outpatient care cases of CMDs and their respective co-twins without CMDs. This enabled us to compare discordant twins while using GBTM. However, we included the cases of CMDs during the follow-up and followed all individuals from 1998 to 2020 without accounting for the timing of the incidence. Thus, the trajectories may have included time before, during and after the CMDs diagnosis. Also, a sensitivity analysis was performed on a sample that included only discordant MZ and DZ same-sex pairs. Since the number and shape of the best-fitting GBTM models were the same as in the final sample, the data limited to MZ/DZ same-sexed twins is not shown.

For the best-fitting trajectory model, we ran multinomial regression models with age, sex, marital status, education, occupational sector and residential region for each trajectory group using the largest trajectory group as a reference. These models were fitted separately for those with and without CMD. Regression models provided the OR and 95% CIs. We tested adding zygosity to the models as both a factor of interest and as a covariate.

## Results

Our final sample of 11,058 twin individuals included 5529 (50%) inpatient and specialised outpatient care cases of CMDs and their respective co-twin within a twin pair without CMDs (table 1). More females (61%) were inpatient and specialised outpatient care cases of CMDs than males (39%). In other baseline characteristics, no clear differences were detected between those with or without CMDs. Out of those without CMDs, 26% had another mental health diagnosis during the follow-up (online supplemental table S1).

**Table 1:** Descriptive characteristics of the final sample (n=11,058)

Baseline (1998)	Inpatient and specialised outpatient care cases of CMDs (n=5529)		Without CMDs (n=5529)	
Sex	**n**	**%**	**n**	**%**
Male	2117	39	2735	49
Female	3412	61	2794	51
Age				
18–24 years	788	16	773	17
25–34 years	1503	27	1329	26
35–44 years	1323	24	1193	24
45–54 years	1284	24	1223	24
55–64 years	485	9	486	10
Education				
<10 years	886	17	880	18
10–12 years	2651	50	2420	48
>12 years	1830	33	1662	32
Missing	16	0	42	1
Marital status				
Married or cohabitant without children	615	11	624	12
Married or cohabitant with children	1442	27	1457	29
Single without children	2887	54	2643	54
Single with children	439	8	280	6
Residential region				
Cities	1922	39	1841	40
Towns and suburbs	2023	41	1825	40
Rural areas	991	20	927	20
Prior musculoskeletal diagnosis	41	1	34	1
Prior mental diagnosis	77	1	91	1

CMDs, common mental disorders.

The trajectory group analysis indicated the best fit for three trajectory group solutions for those inpatient and specialised outpatient care cases of CMDs and two trajectory groups for those without CMDs (table 2).

**Table 2:** Goodness-of-fit statistics of group-based trajectory analysis models

	Non-exposed group—without CMDs				
	Smallest group		BIC	AIC	APP
	N	%			
2-cluster model	**897**	**17**	**−93,655.5**	**−93,626.8**	**0.98**
3-cluster model	910	17	−93,672.9	−93,629.8	0.97
4-cluster model	–	0	−92,971.9	−92,914.5	–
	**Exposed group—inpatient and specialised outpatient care cases of CMDs**				
2-cluster model	1302	24	−94,541.4	−94,512.5	0.98
3-cluster model	**795**	**15**	**−93,526.7**	**−93,483.4**	**0.97**
4-cluster model	104	2	−93,382.6	−93,324.9	0.80
5-cluster model	–	0	−93,379.8	−93,307.6	–

The model presented is shown in bold.

AIC, Akaike Information Criterion; APP, average posterior probability; BIC, Bayesian Information Criterion; CMDs, common mental disorders.

**Figure 2:**
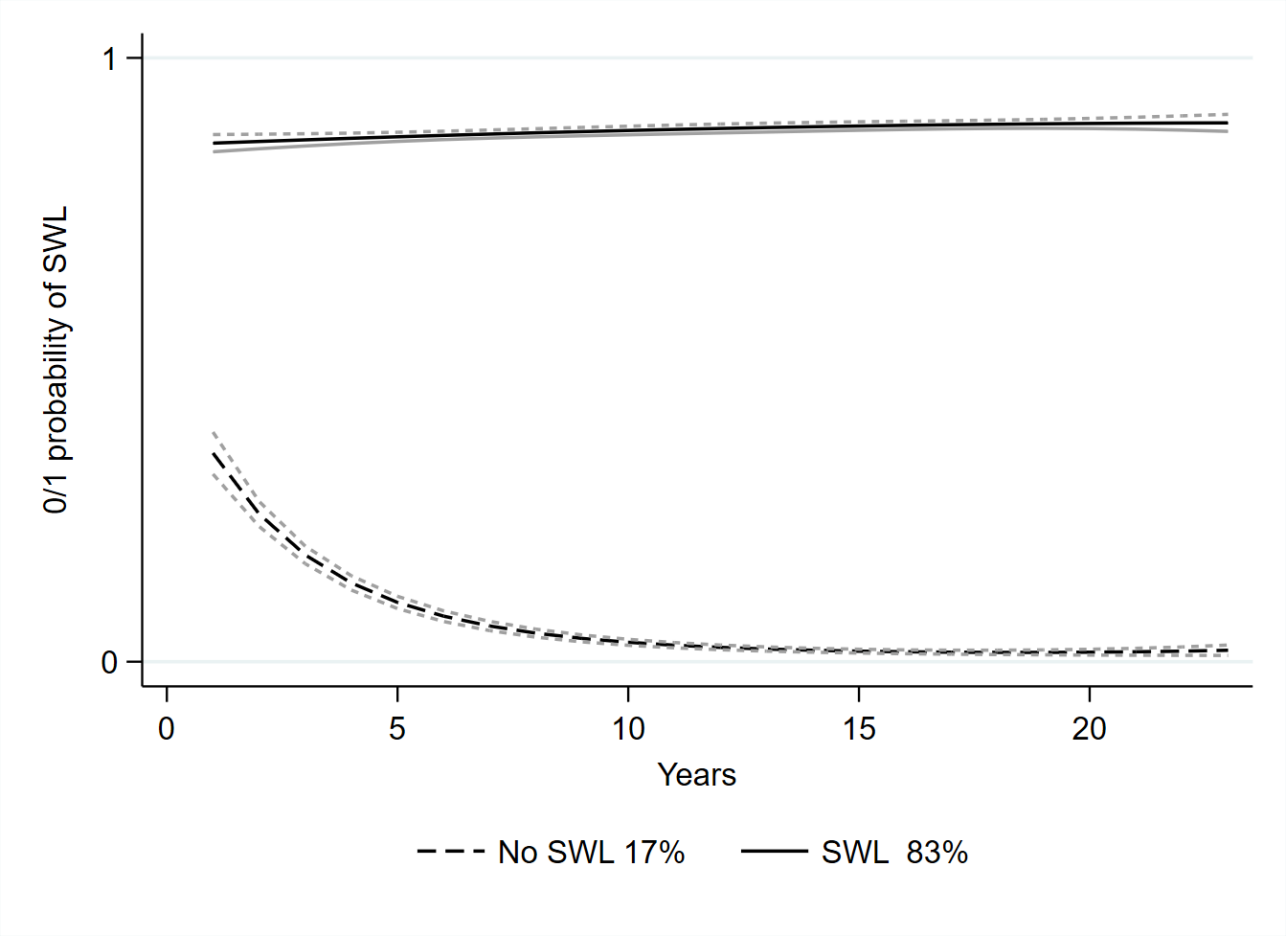
Trajectories of SWL among those without common mental disorders. SWL, sustainable working life.

These cluster groups were named based on their sustainable working life characteristics. For those without CMDs (figure 2), the clusters were:

- cluster 1 (17.0%), no sustainable working life
- cluster 2 (83.0%), sustainable working life

**Figure 3:**
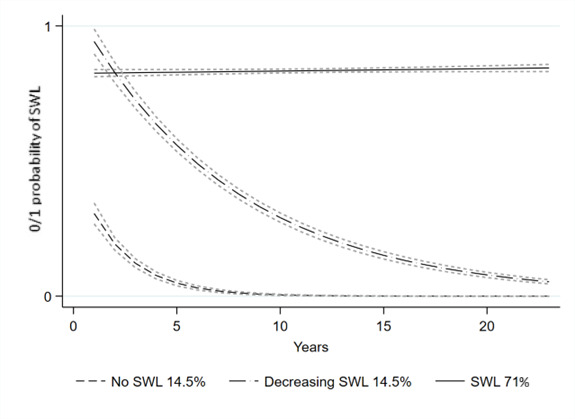
Trajectories of SWL among inpatient and specialised outpatient care cases of common mental disorders. SWL, sustainable working life.

For inpatient and specialised outpatient care cases of CMDs (figure 3), the clusters were:

- cluster 1 (14.5%), no sustainable working life
- cluster 2 (14.5%), decreasing sustainable working life
- cluster 3 (71.0%), sustainable working life

The regression analysis (table 3) indicated that a higher educational level was associated with a lower likelihood of belonging to trajectory groups with no or decreasing SWL, both among those with and without CMDs. Instead, being single (with or without children) was associated with a higher likelihood. Prior mental diagnoses increased the likelihood of belonging to trajectory group 1 among those without CMDs, whereas prior MSD was associated with an increased likelihood of belonging to trajectory group 1 among inpatient and specialised outpatient care cases of CMDs. In the models with zygosity to control the potential effects of familial factors, the point estimates retained the magnitude and direction, so we decided not to show these results.

**Table 3:** Multinomial logistic regression models for ORs and 95% CIs of associations between individual characteristics and trajectory cluster memberships

Baseline (1998)	Inpatient and specialised outpatient care cases of CMDs,* ref cluster 3 (SWL)				Without CMDs,* ref cluster 2	
	Cluster 1 ‘no SWL’		Cluster 2 ‘decreasing SWL’		Cluster 1 ‘no SWL’	
	OR	95% CI	OR	95% CI	OR	95% CI
Sex						
Female (vs male)	**1.26**	**1.03 to 1.54**	0.96	0.81 to 1.14	**1.21**	**1.01 to 1.46**
Education						
<10 years	–	ref	–	ref	–	ref
10–12 years	**0.47**	**0.38 to 0.59**	**0.76**	**0.61 to 0.94**	**0.45**	**0.36 to 0.55**
>12 years	**0.12**	**0.09 to 0.17**	**0.36**	**0.28 to 0.47**	**0.17**	**0.13 to 0.23**
Marital status						
Married or cohabitant without children	–	ref	–	ref	–	ref
Married or cohabitant with children	0.96	0.68 to 1.35	0.79	0.58 to 1.07	**0.7**	**0.50 to 0.98**
Single without children	**2.51**	**1.85 to 3.39**	**1.53**	**1.15 to 2.03**	**2.58**	**1.93 to 3.45**
Single with children	**2.23**	**1.51 to 3.30**	**1.49**	**1.04 to 2.15**	1.53	0.98 to 2.39
Residential region						
Cities	1	ref	–	ref	–	ref
Towns and suburbs	0.98	0.79 to 1.22	1	0.83 to 1.21	0.96	0.78 to 1.18
Rural areas	1.17	0.90 to 1.51	1.04	0.83 to 1.31	0.92	0.72 to 1.18
Prior musculoskeletal diagnosis (vs no)	**3.09**	**1.26 to 7.59**	1.51	0.57 to 3.96	**2.73**	**1.15 to 6.45**
Prior mental diagnosis (vs no)	1.95	0.91 to 4.20	1.49	0.66 to 3.37	**7.04**	**3.50 to 14.14**

* The analyses were adjusted for age and the cohort entry year

CMDs, common mental disorders; ref, reference; SWL, sustainable working life.

## Discussion

This study was based on the population-based sample of 5529 Swedish twin pairs between age 18 and 59 years at the baseline in 1998 and being discordant for the first incident CMDs during the follow-up until 2020 for the trajectories of sustainable working life. The identified trajectories of sustainable working life over time were markedly different for inpatient and specialised outpatient care cases of CMDs or those without CMDs. For those without, we detected two clusters: the one where the majority (83%) had a sustainable working life, whereas 17% had no . For inpatient and specialised outpatient care cases of CMDs, the patterns over time differed as three clusters were detected. The majority (71%) had a sustainable working life, and 14.5% had no sustainable working life, but there was a cluster (14.5%) with decreasing sustainable working life over 23 years of follow-up. Although the onset of CMDs was not accounted for, this indicates that there might be some vulnerable periods. Such periods could have the potential to target actions or interventions to prevent the risk of not having a sustainable working life. Our findings, based on the data-driven GBTM over 23 years and thus being explorative, still add to the previous knowledge based on the SA,^10 11^ return-to-work^12 13^ or a specific occupational sector^14^ and align the findings that CMDs have a major negative influence on work participation.^15 16^

While assessing baseline factors for the associations with trajectory group membership, higher levels of education were universally associated with a lower likelihood of belonging to trajectories with no or decreasing sustainable working life. On the other hand, being single with or without children increased the likelihood of belonging to those. This might imply the importance of socioeconomic factors for sustainable working life, both among inpatient and specialised outpatient care cases of CMDs and those without CMDs, as shown in earlier studies.^21 41^ Furthermore, since prior MSD or prior mental diagnosis was shown to increase the likelihood of belonging to no sustainable working life trajectories, early identification of individuals with health issues and support to remain in working life should be emphasised across all levels, from school healthcare to workplaces and society-level actions. In terms of prevention and action, the trajectory group with 14.5% of inpatient and specialised outpatient care cases of CMDs with decreasing sustainable working life during the follow-up seems a potential target.

The specific feature of our sample, twins, enabled us to shed light on the role of familial factors (ie, genetics and shared, usually childhood and environment) on the trajectories of sustainable working life. A special opportunity was the fact that our sample selection for the first incident CMDs created a design of co-twin control^32^ as we included those without CMDs as a comparison. Hence, this further emphasises that the trajectories were different for inpatient and specialised outpatient care cases of CMDs compared with those without CMDs. As we ran the separate analyses limited to MZ and same-sexed DZ twins, those with and without CMDs were matched for age and sex while controlling for genetics and shared family background (mainly in childhood). The role of familial factors seems not to be the major one—both the trajectories and the regression results remained when assessed among MZ and same-sex DZ twins only. Hence, future studies should investigate the role of familial factors, as these have been indicated to play a role in earlier studies.^2526 28^,^31^ Thus, although the discordance was confirmed for CMDs, 26% of those without CMDs had other mental diagnoses during the follow-up. We cannot rule out that they influenced the identified trajectories, which suggests that further analyses should build on the lifetime discordance for the factor of interest. However, overall, our results based on the trajectories highlight that early identification of individuals with CMDs should be emphasised to react on the first contact with healthcare to ensure the provision of support to remain in working life.

The strengths of this study lie in the relatively large sample, high quality register data without loss to follow-up or recall or reporting biases, and long follow-up time. The use of twins enabled control for familial factors, which have rarely been investigated for sustainable working life and more often for SA/DP.^28^,^31^ Despite the population-based twin data, our results might be less applicable to other countries than the Nordic ones since they share similar welfare and society. Besides this limitation, another might be related to the register data, which does not include symptoms or other health issues besides diagnoses for inpatient or specialised outpatient visits. Our register data on CMDs was based on inpatient or specialised outpatient care, which means that our sample constituted more severe or longer cases of CMDs. Future studies should also include cases in primary healthcare to capture the full picture. On the other hand, the register data included ICD codes for diagnoses set in healthcare, which is a strength compared with self-reported data that is prone to reporting and memory biases. The focus on CMDs can be questioned, as both broader diagnosis groups and a special focus on single diagnoses (eg, depression or anxiety) could have been possible. Future studies could elaborate on these. Furthermore, we would like to acknowledge the fact that the prevalence of severe mental disorders among those without CMDs was 17% (online supplemental table S1). For the results of this study, this means that among the ‘no CMD’ trajectories, there are individuals with severe mental health issues, which further emphasises the fact that most of them had a sustainable working life. Since mental ill-health has an early onset,^4^ special attention should be paid to those without a sustainable working life. We also encourage additional studies to investigate the role of diagnoses even among those without CMDs. We also lacked data on working conditions, such as job quality or work loading, leisure-time activities and individual characteristics such as body mass index. However, since this study was designed as a data-driven exploratory analysis with longitudinal follow-up using a matched discordant twin cohort for identifying the patterns of sustainable working life, such shortcomings could be evaluated in future studies. As we focused on the first incident CMDs, the timing and recurring events of CMDs should be accounted for with different designs. Hence, this calls also for additional studies with modelling that enables tracking the number of episodes, recurrence and/or length of CMDs.

## Conclusion

A majority of inpatient and specialised outpatient care cases of CMDs and those without CMDs had a sustainable working life. Instead, a small cluster among those with CMDs tended to follow a decreasing sustainable working life pattern, and only a minority had no sustainable working life regardless of CMD status. Although a sustainable working life seems prevalent, those with CMDs and with decreased sustainable working life should be identified early for preventive actions and support to remain in working life.

## Supplementary material

10.1136/bmjopen-2025-101586 online supplemental file 1

## Data Availability

Data may be obtained from a third party and are not publicly available.
