# Supplementary material for: The role of common mental disorders on sustainable working life—a cohort study among discordant Swedish twin pairs
Source: BMJ Open. 2025 Nov 4;15(11):e101586. doi: 10.1136/bmjopen-2025-101586 (PMC12587904; doi:10.1136/bmjopen-2025-101586)
Supplement: online supplemental file 1 [file bmjopen-15-11-s001.docx]

**Supplemental table S1** First incident other mental diagnoses for those without CMD

|  | **Without CMD**  **(n = 5529)** | |
| --- | --- | --- |
|  | **n** | **%** |
| Other mental diagnosis |  |  |
| Neuropsychiatric | 127 | 3 |
| Severe mental illness | 164 | 3 |
| Substance use | 604 | 11 |
| Other | 436 | 9 |

| **Supplemental table S2** Frequencies of the latest F-diagnoses before baseline (1998) | | |
| --- | --- | --- |
| **F-diagnosis** | **With CMDs** | **Without CMDs** |
|  | **n** | **n** |
| F10 | 12 | 26 |
| F20 | - | 8 |
| F31 | - | 8 |
| F32 | 21 | 7 |
| F33 | 7 | - |
| F41 | 10 | - |
| F60 | - | 12 |

F06, F11-F19, F22-F29, F34-F40, F43-F51, and F79-F98 had frequency ≤5 for both with or without CMDs; F20, F31 and F60 had frequency ≤5 for those with CMDs; and F33 and F41 ≤5 for those without CMDs
