# Supplementary material for: The role of common mental disorders on sustainable working life—a cohort study among discordant Swedish twin pairs
Source: BMJ Open. 2025 Nov 4;15(11):e101586. doi: 10.1136/bmjopen-2025-101586 (PMC12587904; doi:10.1136/bmjopen-2025-101586)
Supplement: Supplementary file 2 [file bmjopen-2025-101586.reviewer_comments.pdf]

PEER REVIEW HISTORY

BMJ Open publishes all reviews undertaken for accepted manuscripts. Reviewers are asked to complete a checklist review form and are provided with free text boxes to elaborate on their assessment. These free text comments are reproduced below.

ARTICLE DETAILS

Title (Provisional)

The role of common mental disorders on sustainable working life - a cohort study among discordant Swedish twin pairs

Authors

Ropponen, Annina; Alaie, Iman; Narusyte, Jurgita; Svedberg, Pia

---

VERSION 1 - REVIEW

**Reviewer**	**1**
**Name**	**Dewi, Ayu Putu Madri**
**Affiliation**	**Amsterdam UMC Location AMC, Epidemiology and Data Science**
**Date**	**02-Apr-2025**
**COI**	**None**

Manuscript Title:

The role of common mental disorders on sustainable working life - a cohort study among discordant Swedish twin pairs

Dear authors,

I am an Open Science researcher, who has checked your manuscript for its adherence to a number of Open Science practices.

Below you find the checklist that I used to assess your manuscript for adherence to a number of these practices.

Based on this assessment, I would suggest the following refinements/additions:

1. Availability of a protocol: Please provide a link to the protocol.

2. Pre-registration: Please provide information on whether and where the study was pre-registered.

3. Open Materials: N/A

4. Open Data: Given the specific restrictions apply to availability of the data used in the study, please specify whether de-identified, metadata, or a subset of the data can still be made publicly available.

5. Open Code: Please provide (a link to) the code used for the analyses in this study.

6. Citations: Please provide citations for your materials/data/codes.

7. Software and tools used are described: OK

8. Open access publication: OK

9. Reporting checklist provided: OK

10. Preprint: Please state explicitly whether and where the preprint of this study can be found.

**Reviewer**	**2**
**Name**	**Dickens, Geoffrey**
**Affiliation**	**Northumbria University, Nursing Midwifery and Health**
**Date**	**14-May-2025**
**COI**	**None**

This was a well-conducted study reported in accordance with STROBE guidelines. The study draws on national register data from Sweden which appears quite comprehensive. The question seems important since higher levels of worklife interruption in people with common mental disorders might potentially be ameliorated. A small number of clarifications are required.

Abstract: Strengths and limitations bullet 4 "The first incident…" This statement seems opaque. Presumably it refers to the fact that the onset of CMD may precede or follow the onset of significant disruption to working life. In effect the causality of the relationship between CMD and worklife disruption cannot be established. This should be more clearly stated.

P12 Line 46 "The regression analysis (Table 3) indicated that higher educational level was associated with less likelihood of belonging to all trajectory groups both among those with and without CMDs." This seems poorly worded – is it not that higher education was associated with less likelihood of belonging to "SWL" cluster in all other trajectory groups?

Follow up age ended at 65 Presumably this being retirement age in both M and F in Sweden please clarify.

**Reviewer**	**3**

**Name**	**Porru, Fabio**
**Affiliation**	**Erasmus Medical Center, Public Health**
**Date**	**09-Jun-2025**
**COI**	**None**

Dear Authors, we read with great interest your work. We were extremely curious to see your application of trajectory analysis on such rich dataset. We believe your work has the potential to contribute significantly to the literature in the field of work participation, and in particular to better understand the role of mental disorders in decreasing work participation. Nonetheless, there are major problems we believe you should address to unlock the true potential of this study and with our feedback, focused on introduction and methods, we truly hope to help you in doing so.

Introduction

The introduction needs to (and can) be improved significantly.

1. The rationale in the introduction is not properly summarized. There are multiple statements not backed up properly with findings from the literature in the text. It stays unclear what specific findings are already well-established in the literature and which gaps remain. Providing a brief summary of the key results when referencing previous studies would help the reader to better understand the current state of research and how this study may contribute to it.

2. The aims stated at the end of the introduction are vague. By reading them, we are unable to know what to expect in terms of analysis plan. More space should be devoted to introduce other methodological aspects, such as the use of data register and the trajectory analysis. Also, you dedicate quite some space to discussing the inclusion of twins in this study. Nonetheless, it is not very clear the practical benefits of this in pursuing the research goals of this study. Which gaps from the literature would be fill by this? The introduction should explain the reader how this study would improve what we know about this topic, referring to both contents and methods of the study. Try to be more specific. In some cases, we understand what you may be referring to, but we are not actually sure.

Methods:

The methods section needs to be re-organized, and additional information is needed.

3. Right now you describe study design, population and variables at once. Please, divide and organize the section under appropriate headings to help the reader to navigate your work and your choices:

a. Study design

b. Study population: describe here inclusion/exclusion with rationale for every step. What missing information was considered for exclusion? According to your table, there are individuals with missing information on education level.

c. Measurements: for each variable, provide definition, source, assessment, operationalization of the variables, with rationale. Currently, this information is disorganized.

4. Sustainable working life

a) The inclusion of old-age pension as a non-sustainable working life status warrants further justification. Since eligibility for old-age pension is typically tied to national statutory retirement age, this transition is often voluntary and age-appropriate, rather than a reflection of reduced work capacity or an unsustainable working life. Grouping it with sickness absence, disability pension, and unemployment may conflate fundamentally different exit pathways. So if in a given year the statutory retirement age was below 65, and people therefore received old-age pension, it is now considered the same as when someone is in sick leave or receives disability pension?

b) Can you provide your rationale of using the cutoff of 6-months. Is it based on previous literature, data-driven, legislation, or what? We may argue that a cutoff of 6 months may be inadequate as it would consider "sustainable" someone working for 6 months and one day out of 12 months. In this frame, is "sustainable" the best choice as term for this variable? Did you consider talking about work participation?

5. Mental disorders

a. You are using a classification system that many in the literature use when only less detailed data are available. Why do you use "common" mental disorders rather than all mental disorders? If you want to focus on specific conditions, your data allow you to do this with higher precision. Then, why don't you focus on depressive disorder, anxiety disorder and so on?

b. Another implication of such use of your data on mental disorders is that excluding severe diagnosis implies that people with non-common mental disorders (e.g., schizophrenia) is used as comparison. Rather than addressing this in the article or in the supplementary file, you can make better choices.

6. Other variables: the variables used in the tables differ from those described in the methods. Please, for every variable, write down in the methods the source, how they were assessed and used in the study. Additionally, make sure you use the correct labels for sensitive variables: if your information is sex, as it is likely to be if you use registered data, then the groups should be "male" and "female".

7. Although you are using existing data, you need to provide additional information about ethic details.

Statistical analysis

8. You are using trajectory analysis: why are you using this approach? You may link this also to the comment concerning the introduction. We could personally imagine using this data with other approaches (e.g., survival analysis with Cox regression). Help the readers to understand your choice.

9. You do not take into account the age of diagnosis. Setting baseline at the time of CMD diagnosis would make more methodological and conceptual sense given the study's aim: which is to investigate patterns of sustainable working life among individuals with CMDs and to examine familial and sociodemographic factors influencing those patterns.

10. It may not be appropriate to use a linear polynomial model as the default specification in this context, although I acknowledge this is based on my expectations of the data. Given the nature of the outcome: employment-related status over a long follow-up period, it seems unlikely that individuals would follow a strictly linear trend. Employment trajectories often involve fluctuations, such as transitions in and out of work, periods of sickness absence, or early retirement. A more flexible functional form, such as a quadratic or cubic polynomial, may better capture these non-linear patterns. I would suggest either justifying the choice of a linear model or testing alternative specifications if feasible.

11. The selection of the optimal model is inconsistent. If you apply the methods described above to elect the optimal number and shape of trajectories, in both cases the best fit statistics are found in the 2 cluster model. The goal of GBTM the goal is not solely to achieve optimal statistical model fit, but rather to find a balance between model fit and interpretability that aligns with the study's research aims. However, transparency is warranted.

VERSION 1 - AUTHOR RESPONSE

Reviewer: 1
Ms. Ayu Putu Madri Dewi, Amsterdam UMC Location AMC
Comments to the Author:
Manuscript Title: The role of common mental disorders on sustainable working life - a cohort study among discordant Swedish twin pairs

Dear authors,
I am an Open Science researcher, who has checked your manuscript for its adherence to a number of Open Science practices.
Below you find the checklist that I used to assess your manuscript for adherence to a number of these practices.
Response: Thank you for providing these for us.

Based on this assessment, I would suggest the following refinements/additions:

1. Availability of a protocol: Please provide a link to the protocol.

Response: There is no pre-registered protocol, but the study, including aims, methods, and analyses, is described in detail in the manuscript. We would like to acknowledge the appreciation of the initiatives to publish protocols, but since that was not required by the journal or the funding sources of our study, that has not been done.

2. Pre-registration: Please provide information on whether and where the study was pre-registered.

Response: There is no pre-registered protocol.

3. Open Materials: N/A

Response: Based on N/A, we assume this does not apply to us.

4. Open Data: Given the specific restrictions apply to availability of the data used in the study, please specify whether de-identified, metadata, or a subset of the data can still be made publicly available.

Response: As stated in our Availability of data and material statement, the data (even the parts of it) that support the findings of this study are available from the original sources: the Swedish Twin Registry, Statistics Sweden, Swedish Social Insurance Agency, and the Swedish National Board of Health and Welfare. Restrictions apply to the availability of the data used in this study based on the Swedish Twin project Of Disability pension and Sickness absence (STODS), which were used with ethical permission for the current study and therefore are not publicly available.

5. Open Code: Please provide (a link to) the code used for the analyses in this study.

Response: Due to the restrictions in data availability, we have not prepared the code for the analyses to be publicly available.

6. Citations: Please provide citations for your materials/data/codes.

Response: All relevant citations have been provided within the limits of the allowed number of references by the journal.

7. Software and tools used are described: OK
8. Open access publication: OK
9. Reporting checklist provided: OK

Response: Based on the OKs in points 7-9, we assume they are not requested from us.

10. Preprint: Please state explicitly whether and where the preprint of this study can be found.

Response: No preprint of this study has been published.

Reviewer: 2
Dr. Geoffrey Dickens, Northumbria University
Comments to the Author:
This was a well-conducted study reported in accordance with STROBE guidelines. The study draws on national register data from Sweden which appears quite comprehensive. The question seems important since higher levels of worklife interruption in people with common mental disorders might potentially be ameliorated. A small number of clarifications are required.

Response: We appreciate this positive feedback.

Abstract: Strengths and limitations bullet 4 "The first incident…" This statement seems opaque. Presumably it refers to the fact that the onset of CMD may precede or follow the onset of significant disruption to working life. In effect the causality of the relationship between CMD and worklife disruption cannot be established. This should be more clearly stated.

Response: We revised the bullet to read: "**A population-based sample of 18-59-year-old Swedish-born twins discordant for the common mental disorders was followed from the baseline in 1998 until 2020.**"

P12 Line 46 "The regression analysis (Table 3) indicated that higher educational level was associated with less likelihood of belonging to all trajectory groups both among those with and without CMDs." This seems poorly worded – is it not that higher education was associated with less likelihood of belonging to "SWL" cluster in all other trajectory groups?
Response: In Table 3, Cluster 3 "SWL" is the reference group that all other trajectory groups are compared to. Thus, our finding that higher education yields OR < 1 indicates that with higher education, they are less likely to belong to "No SWL" or "Decreasing SWL" groups, regardless of the CMDs status. However, to add clarity to this sentence, it has been rephrased and now reads: "The regression analysis (Table 3) indicated that a higher educational level was associated with a lower likelihood of belonging to trajectory groups **with no or decreasing SWL,** both among those with and without CMDs."

Follow up age ended at 65 Presumably this being retirement age in both M and F in Sweden please clarify.
Response: There is no statutory pension age in Sweden. However, most people retire at the age of 65, so this age was applied as an ending point.

Reviewer: 3
Dr. Fabio Porru, Erasmus Medical Center, University of Cagliari
Comments to the Author:
Dear Authors, we read with great interest your work. We were extremely curious to see your application of trajectory analysis on such rich dataset. We believe your work has the potential to contribute significantly to the literature in the field of work participation, and in particular to better understand the role of mental disorders in decreasing work participation. Nonetheless, there are major problems we believe you should address to unlock the true potential of this study and with our feedback, focused on introduction and methods, we truly hope to help you in doing so.
Response: We appreciate your kind words.

Introduction
The introduction needs to (and can) be improved significantly.
1. The rationale in the introduction is not properly summarized. There are multiple statements not backed up properly with findings from the literature in the text. It stays unclear what specific findings are already well-established in the literature and which gaps remain. Providing a brief summary of the key results when referencing previous studies would help the reader to better understand the current state of research and how this study may contribute to it.

Response: We have revised the introduction as suggested by adding further details and interpretations. We have also reasoned our statistical approach in the introduction based on a later comment by this reviewer.

2. The aims stated at the end of the introduction are vague. By reading them, we are unable to know what to expect in terms of analysis plan. More space should be devoted to introduce other methodological aspects, such as the use of data register and the trajectory analysis. Also, you dedicate quite some space to discussing the inclusion of twins in this study. Nonetheless, it is not very clear the practical benefits of this in pursuing the research goals of this study. Which gaps from the literature would be fill by this? The introduction should explain the reader how this study would improve what we know about this topic, referring to both contents and methods of the study. Try to be more specific. In some cases, we understand what you may be referring to, but we are not actually sure.

Response: We have paid special attention to these parts of the introduction and aims on pages 4-6, trying to capture the suggestions.

Methods:
The methods section needs to be re-organized, and additional information is needed.
3. Right now you describe study design, population and variables at once. Please, divide and organize the section under appropriate headings to help the reader to navigate your work and your choices:
a. Study design
b. Study population: describe here inclusion/exclusion with rationale for every step. What missing information was considered for exclusion? According to your table, there are individuals with missing information on education level.
c. Measurements: for each variable, provide definition, source, assessment, operationalization of the variables, with rationale. Currently, this information is disorganized.

Response: We have modified these sections as suggested and used the subheadings provided by the reviewer.

4. Sustainable working life
a) The inclusion of old-age pension as a non-sustainable working life status warrants further justification. Since eligibility for old-age pension is typically tied to national statutory retirement age, this transition is often voluntary and age-appropriate, rather than a reflection of reduced work capacity or an unsustainable working life. Grouping it with sickness absence, disability pension, and unemployment may conflate fundamentally different exit pathways. So if in a given year the statutory retirement age was below 65, and people therefore received old-age pension, it is now considered the same as when someone is in sick leave or receives disability pension?

Response: The reviewer is right that the old-age pension might be an issue here. However, we utilized the age of 65 as the end of the follow-up. There is no statutory pension age in Sweden, but most will retire at the age 65 years of age during the follow-up years in this study. Thus, when assigning the individual to the yearly observations, death was given priority over old-age pension, and old-age pension was given priority over SA, DP, and unemployment. Since death, obviously, is a non-return, it was assigned first, meaning that if the individual died during the year or was already dead at the beginning of the year, she or he was censored. Then, in case of those older individuals, an old-age pension was assigned if the individual had more than half of their yearly income from the

old-age pension during the year. Thus, the old-age pension could only affect the end of follow-up at age 65.

b) Can you provide your rationale of using the cutoff of 6-months. Is it based on previous literature, data-driven, legislation, or what? We may argue that a cutoff of 6 months may be inadequate as it would consider "sustainable" someone working for 6 months and one day out of 12 months. In this frame, is "sustainable" the best choice as term for this variable? Did you consider talking about work participation?

Response: Using the 6-month cut-off was based on earlier work in Sweden using similar register data to ours. See e.g., Norberg J, Alexanderson K, Framke E, Rugulies R, Farrants K. Job demands and control and sickness absence, disability pension and unemployment among 2,194,692 individuals in Sweden. Scand J Public Health. 2020 Mar;48(2):125-133. doi: 10.1177/1403494819846367. Furthermore, we have utilized this 6-month cut-off in our series of papers about sustainable working life and ended up using this wording based on a definition paper with a systematic literature review: Ropponen A, Wang M, Narusyte J, Silventoinen K, Böckerman P, Svedberg P. Sustainable Working Life in a Swedish Twin Cohort-A Definition Paper with Sample Overview. Int J Environ Res Public Health. 2021 May 28;18(11):5817. doi: 10.3390/ijerph18115817. We agree with the reviewer that other cut-offs or wordings could be utilized, but for comparability in our studies, we would like to retain that. For the reviewer's convenience, some of the papers from our series are listed below (and above):

Ropponen A, Wang M, Raza A, Narusyte J, Svedberg P. Night Work and Sustainable Working Life-A Prospective Trajectory Analysis of Swedish Twins. Int J Environ Res Public Health. 2022 Aug 31;19(17):10857. doi: 10.3390/ijerph191710857.

Ropponen A, Wang M, Alaie I, Narusyte J, Svedberg P. Concurrent trajectories of residential region in relation to a sustainable working life among Swedish twins. Eur J Public Health. 2023 Aug 1;33(4):596-600. doi: 10.1093/eurpub/ckad053.

Ropponen A, Narusyte J, Wang M, Silventoinen K, Böckerman P, Svedberg P. Genetic and environmental contributions to individual differences in sustainable working life-A Swedish twin cohort study. PLoS One. 2023 Jul 27;18(7):e0289074. doi: 10.1371/journal.pone.0289074.

Ropponen A, Bergström J, Narusyte J, Svedberg P. A life course study of genetic and environmental influences on sustainable working life. PLoS One. 2025 Feb 25;20(2):e0317675. doi: 10.1371/journal.pone.0317675.

5. Mental disorders
a. You are using a classification system that many in the literature use when only less detailed data are available. Why do you use "common" mental disorders rather than all mental disorders? If you want to focus on specific conditions, your data allow you to do this with higher precision. Then, why don't you focus on depressive disorder, anxiety disorder and so on?

Response: We appreciate that the reviewer can see the potential of our data. However, given the fact that all diagnoses in F0-F99 represent a broad heterogeneity, we also assumed that focusing on separate or single diagnosis groups (such as depression or anxiety, as suggested by the reviewer) could add complexity to analyses or even merit separate papers. Yet another fact is that, e.g., depression and anxiety share the etiology to a large extent while also being highly comorbid. Furthermore, as can be seen from Figure 1, our sample size diminished considerably from the

original (around 120,000 individuals) to the final sample of 5529 individuals with CMD. Thus, further studies with either different inclusion criteria or even larger sample sizes would be merited to investigate more detailed diagnoses. To acknowledge this, we added a sentence to the discussion: "**The focus on CMDs can be questioned as both broader diagnostic groups and a special focus on single diagnoses (e.g., depression or anxiety) could have been possible. Further studies could elaborate on these.**"

b. Another implication of such use of your data on mental disorders is that excluding severe diagnosis implies that people with non-common mental disorders (e.g., schizophrenia) is used as comparison. Rather than addressing this in the article or in the supplementary file, you can make better choices.

Response: We acknowledge this concern. However, we would like to pinpoint that the number of those severe mental diagnoses in the "no CMD" group was relatively low, 17% (see the Supplemental Table S1). Furthermore, due to the data-driven approach using GBTM, excluding further individuals from the analytical sample would mean analyzing the whole data set again. Thus, the suggestions to test any other solutions for diagnosis categorizations (we also refer to the earlier comment about our focus on CMDs) would best be done in dedicated, further studies with specific aims to elaborate on the role of single diagnosis groups. Thus, after careful consideration, we have decided not to test further solutions in this paper but will consider them in the future.

6. Other variables: the variables used in the tables differ from those described in the methods. Please, for every variable, write down in the methods the source, how they were assessed and used in the study. Additionally, make sure you use the correct labels for sensitive variables: if your information is sex, as it is likely to be if you use registered data, then the groups should be "male" and "female".

Response: Thank you for pointing out these discrepancies. They have now been corrected, and we added the relevant references while also following your suggestion to use female/male instead of men/women.

7. Although you are using existing data, you need to provide additional information about ethic details.

Response: Ethics is a top-priority issue in all studies. Without having further precision on the reviewer's suggestion, we would like to note that we have provided the "Ethics approval and consent to participate" as requested by the journal. It reads: "The study protocol was designed and performed according to the principles of the Helsinki Declaration. The ethical vetting was performed and approved by the Regional Ethical Review Board of Stockholm, Sweden (Dnr: 2007/524-31, 2010/1346-32/5, 2014/311-32, 2015/1809-32, 2017/128-32). For this project, the Regional Ethical Review Board of Stockholm stated that the consent to participate was not applicable in these types of large register studies." If further amendments are needed, please provide us with the request.

Statistical analysis
8. You are using trajectory analysis: why are you using this approach? You may link this also to the comment concerning the introduction. We could personally imagine using this data with other approaches (e.g., survival analysis with Cox regression). Help the readers to understand your choice.

Response: We agree with the reviewer that many other statistical approaches could have been chosen for the analyses. However, along with the earlier suggestion by the reviewer to make the caveats of the knowledge clearer in our introduction, we have also amended the reasoning why GBTM was utilized on pages 4-5. On page we added: "**Thus, we should gain further knowledge of**

the developmental, longitudinal patterns of working life participation across a life course to compare those with and without CMDs, and use an approach that enables us to approximate the proportion of individuals following specific trajectories over time [16] " and on page 5: "In this study, we aimed to investigate patterns of sustainable working life among Swedish twins discordant for CMDs **using detailed register data with group-based trajectory modeling**. Another aim was to estimate the role of baseline sociodemographic and familial factors for the **data-driven** identified trajectory groups."

9. You do not take into account the age of diagnosis. Setting baseline at the time of CMD diagnosis would make more methodological and conceptual sense given the study's aim: which is to investigate patterns of sustainable working life among individuals with CMDs and to examine familial and sociodemographic factors influencing those patterns.

Response: We are aware that the age of diagnosis is missing from our analysis. That might be a factor to be considered in future studies. Since our data consisted of twins, our discordant twin pairs were age and sex matched by default. That does not remove the fact that the age of diagnosis was missing, but it adds rigor to the comparison of those with and without CMDs. Furthermore, we would like to note that accounting for the age of diagnosis might have required a different statistical data approach than the current one, thus making it appealing for future studies to consider that fact. Another alternative would be to use the age as the condition followed over time, instead of time as we did. However, that would require a specific research question, not the one we have stated in our aims. All in all, even without the age of diagnosis, our developmental trajectories indicate the sustainable working life patterns across the life course for those with CMDs, even before, during, and after, compared to their matched pairs without.

10. It may not be appropriate to use a linear polynomial model as the default specification in this context, although I acknowledge this is based on my expectations of the data. Given the nature of the outcome: employment-related status over a long follow-up period, it seems unlikely that individuals would follow a strictly linear trend. Employment trajectories often involve fluctuations, such as transitions in and out of work, periods of sickness absence, or early retirement. A more flexible functional form, such as a quadratic or cubic polynomial, may better capture these non-linear patterns. I would suggest either justifying the choice of a linear model or testing alternative specifications if feasible.

Response: Model specifications are always at the core of the statistical analyses. The reviewer nicely summarized the options provided by the GBTM. While performing the original analyses, we tested the various model specifications before obtaining the final results with the linear polynomial model. As the Average Posterior Probability Assignment (APPA) and Odds of correct classification (OCC) pointed towards the linear model (APPA being >70% and OCC being >5.0 for each class), that was chosen following the recommendations reported for GBTM. We have added this in our statistical methods on page 8 with a relevant reference.

11. The selection of the optimal model is inconsistent. If you apply the methods described above to elect the optimal number and shape of trajectories, in both cases the best fit statistics are found in the 2 cluster model. The goal of GBTM the goal is not solely to achieve optimal statistical model fit, but rather to find a balance between model fit and interpretability that aligns with the study's research aims. However, transparency is warranted.

Response: We agree with the reviewer that model selection is always based on both the statistical fit, study aims, and even on visual inspection of trajectories. We would like to emphasize that we have followed this protocol with a light emphasis on BIC fit indexes and therefore considered that the 2-cluster model for those without CMD and the 3-cluster model for those with CMD were best. We added this decision criterion to the statistical analysis section on page 8.

VERSION 2 - REVIEW

**Reviewer**	**3**
**Name**	**Porru, Fabio**
**Affiliation**	**Erasmus Medical Center, Public Health**
**Date**	**23-Sep-2025**
**COI**	

Dear colleagues,

I read once again with interest this work, and I want to inform you that, once again. I reviewed it with a PhD candidate. We read the response of the authors, and we provide here our remarks. The article has improved, but we recommend at least a few more changes before publication. On a couple of matters, we disagree with the authors but we "agree to disagree" as long as they provide clear and transparent information on their rationale, on the implications of their choices (including the limitations) and on how future research may contribute to overcome such limitations.

• Point 1. The background needs additional improvements. There are several sentences unclear, and the narration can be significantly improved.

Two examples from the first two sentences.

"Mental ill-health rates have been increasing in Sweden during the last decade 1 and remain high globally, with approximately 15% of working-age adults having a mental disorder 2."

Where? In Sweden or globally?

This sentence is totally unclear: "These rates also apply to common mental disorders (CMDs), which constitute depressive, anxiety, and stress-related disorders 3, while also reducing work capacity may pose to CMDs."

What rates? First, no rates are listed. Second, you are saying that mental disorders are highly prevalent and then stating that this applies to… mental disorders? Third, the work capacity is coming out of nowhere.

This sentence if quite long and provides a broad set of evidence: ". In the long run, CMDs can significantly reduce an individual's work capacity and stand for most sickness absences (SA), although many people with CMDs are in paid work and are likely to benefit from working 7."

I recommend devoting a bit of time to design a clearer structure and improve the readability and introduce properly the research questions of this study.

• Point 3. In the section "study population", you now include source of data. Please, start this section by clearly defining your study population, and the inclusion/exclusion process. Then, consider moving the sources of each variable in the relative paragraph of the measurements section.

• You excluded cases with missing cases… in what variables?

• Point 4a. Information about the Swedish context is valuable for international readers. Consider providing a couple of sentences to explain what you just explained in response to our comment.

• Point 4b. The rationale of the choice of six-month follow-up is now clear and should be added to the manuscript. This is a valuable information and including it improves the transparency and reproducibility of your work.

• Point 5. I personally do not agree with the authors on this specific matter and I believe this is a missed opportunity, given the availability of the data. Nonetheless, I fully acknowledge that any approach comes with pros and cons. Moreover, changing this coding would require many efforts that our colleagues and they can contribute to the literature by devoting their time elsewhere. Nonetheless, I would like them at least to add in the manuscript i) their rationale in support of this approach in the methods section, ii) a speculation for possible consequences for the results in the discussion. For example, you mention that the prevalence of severe mental disorders is 17%, which is quite high. How may this influence the results?

• Point 10. Please, add and discuss the fit statistics of alternative models for transparency. We strongly recommend adding a rational for every decision in the analysis to improve the reproducibility of the work.

VERSION 2 - AUTHOR RESPONSE

Reviewer: 3
Dr. Fabio Porru, Erasmus Medical Center, University of Cagliari
Comments to the Author:
Dear colleagues,
I read once again with interest this work, and I want to inform you that, once again. I reviewed it with a PhD candidate. We read the response of the authors, and we provide here our remarks. The article has improved, but we recommend at least a few more changes before publication. On a couple of matters, we disagree with the authors but we "agree to disagree" as long as they provide clear and transparent information on their rationale, on the implications of their choices (including the limitations) and on how future research may contribute to overcome such limitations.

Response: We appreciate your feedback and also are delighted that you have provided a PhD candidate with an important learning opportunity.

• Point 1. The background needs additional improvements. There are several sentences unclear, and the narration can be significantly improved.
Two examples from the first two sentences.
"Mental ill-health rates have been increasing in Sweden during the last decade 1 and remain high globally, with approximately 15% of working-age adults having a mental disorder 2."
Where? In Sweden or globally?
This sentence is totally unclear: "These rates also apply to common mental disorders (CMDs), which constitute depressive, anxiety, and stress-related disorders 3, while also reducing work capacity may pose to CMDs."
What rates? First, no rates are listed. Second, you are saying that mental disorders are highly prevalent and then stating that this applies to… mental disorders? Third, the work capacity is coming out of nowhere.
This sentence if quite long and provides a broad set of evidence: ". In the long run, CMDs can significantly reduce an individual's work capacity and stand for most sickness absences (SA), although many people with CMDs are in paid work and are likely to benefit from working 7."
I recommend devoting a bit of time to design a clearer structure and improve the readability and introduce properly the research questions of this study.
Response: Thank you for the detailed suggestions. We have revised the introduction accordingly and paid special attention to the text in other sections as well.

• Point 3. In the section "study population", you now include source of data. Please, start this section by clearly defining your study population, and the inclusion/exclusion process. Then, consider moving the sources of each variable in the relative paragraph of the measurements section.
Response: Done as suggested.

• You excluded cases with missing cases… in what variables?
Response: We added on page 6 "… **in any outcomes or factors of interest.**"

• Point 4a. Information about the Swedish context is valuable for international readers. Consider providing a couple of sentences to explain what you just explained in response to our comment.
Response: We added at the end of page 7: "**We utilized the age of 65 as the end of the follow-up because in Sweden, there is no statutory pension age. However, most will retire at the age of 65."**

• Point 4b. The rationale of the choice of six-month follow-up is now clear and should be added to the manuscript. This is a valuable information and including it improves the transparency and reproducibility of your work.

Response: Thank you for this. This was amended on page 7.

• Point 5. I personally do not agree with the authors on this specific matter and I believe this is a missed opportunity, given the availability of the data. Nonetheless, I fully acknowledge that any approach comes with pros and cons. Moreover, changing this coding would require many efforts that our colleagues and they can contribute to the literature by devoting their time elsewhere. Nonetheless, I would like them at least to add in the manuscript i) their rationale in support of this approach in the methods section, ii) a speculation for possible consequences for the results in the discussion. For example, you mention that the prevalence of severe mental disorders is 17%, which is

quite high. How may this influence the results?

Response: We appreciate this consideration and agree with the reviewer that potential solutions would be many. However, for this manuscript, we added the reasoning of focusing on CMDs on page 6 as the response for i): "**CMDs were chosen since all diagnoses in F0-F99 represent a broad heterogeneity, while depression and anxiety largely share the etiology, and are highly comorbid [33]**." Then, for ii) we amended the discussion on page 15 with "**Furthermore, we would like to acknowledge the fact that the prevalence of severe mental disorders among those without CMDs was 17% (Supplemental Table S1). For the results of this study, this means that among the "no CMD" trajectories, there are individuals with severe mental health issues, which further emphasizes the fact that most of them had a sustainable working life. Since mental ill-health has an early onset [4], special attention should be paid to those without a sustainable working life. We also encourage further studies to investigate the role of diagnoses even among those without CMDs.** "

• Point 10. Please, add and discuss the fit statistics of alternative models for transparency. We strongly recommend adding a rational for every decision in the analysis to improve the reproducibility of the work.

Response: We appreciate this suggestion. However, presentation of the models that are not used as a basis of this study, given that this is not a statistical paper, would add unnecessary reporting and length to this manuscript. Thus, even though we can see the point made by the reviewer, we would kindly like to decline this suggestion as we are following the standard protocol as suggested by the references in our methods section. Furthermore, we would like to pinpoint the reviewer to the earlier papers published in this journal with similar reporting. Please note that we chose only a few of the latest examples:

Li H, Duan J, Chen J, et al. Association between long-term atherogenic index of plasma and carotid atherosclerosis: a prospective cohort study of a health examination population. BMJ Open 2025;15:e098874. doi: 10.1136/bmjopen-2025-098874

Hyun D, Ahn JH, Gil H, et al. Longitudinal trajectories of sedation level and clinical outcomes in patients who are mechanically ventilated based on a group-based trajectory model: a prospective, multicentre, longitudinal and observational study in Korea. BMJ Open 2023;13:e072628. doi: 10.1136/bmjopen-2023-072628

Malaju MT, Alene GD, Azale T. Impact of maternal morbidities on the longitudinal health-related quality of life trajectories among women who gave childbirth in four hospitals of Northwest Ethiopia: a group-based trajectory modelling study. BMJ Open 2022;12:e057012. doi: 10.1136/bmjopen-2021-057012.
